# Impact of preoperative anxiety and depressive symptoms on clinical outcomes of anterior talofibular ligament reconstruction surgery

**DOI:** 10.3389/fpsyt.2025.1660886

**Published:** 2026-01-22

**Authors:** Yong Duan, JunKui Xu, HongXing Zhang, Chi Shang, RuiZe Yang

**Affiliations:** Honghui Hospital, Xi’an Jiaotong University, Xi’an, Shaanxi, China

**Keywords:** anxiety, depression, psychological factors, anterior talofibular ligament (ATFL), ligament repair, clinical outcomes, prognosis

## Abstract

**Purpose:**

This study aimed to compare the clinical outcomes of patients undergoing anterior talofibular ligament (ATFL) repair with or without preoperative anxiety/depressive symptoms, both preoperatively and at the final follow-up, and to quantify the association between these symptoms and the degree of improvement in surgical efficacy.

**Methods:**

A total of 91 patients who underwent ATFL repair at Xi’an Honghui Hospital between January 2015 and August 2023 were included. Baseline characteristics were collected, and assessments were performed using the Hospital Anxiety and Depression Scale (HADS), the American Orthopedic Foot and Ankle Society (AOFAS) Ankle-Hindfoot Scale, and the Visual Analog Scale (VAS), both preoperatively and at the final follow-up. Patients were divided into two groups based on the presence or absence of preoperative anxiety/depressive symptoms: the Anxiety/Depression group (Group A) and the Non-Anxiety/Depression group (Group B). Baseline characteristics such as gender, age, disease duration, BMI, and follow-up duration were statistically analyzed to ensure comparability between groups. Clinical outcomes and changes in evaluation scores between the two time points were then compared.

**Results:**

A total of 81 patients completed the full follow-up, among whom 40 patients (49.38%) presented with preoperative anxiety/depressive symptoms. At the final follow-up, all patients demonstrated significant improvements in VAS, AOFAS, HADS-A, and HADS-D scores compared to preoperative values (P < 0.001). Further analysis revealed that Group B (without preoperative symptoms) had significantly better VAS, AOFAS, HADS-A, and HADS-D scores both preoperatively and postoperatively than Group A (P < 0.001). However, the improvements in HADS-A and HADS-D scores from preoperative to final follow-up were significantly greater in Group A (P < 0.001), whereas no significant differences were observed between groups in the improvement of VAS and AOFAS scores (P > 0.05).

**Conclusions:**

Patients with preoperative anxiety/depressive symptoms demonstrated worse clinical evaluations both preoperatively and at follow-up compared to those without such symptoms. However, both groups experienced comparable improvements in pain and function, while patients with preoperative psychological symptoms showed greater mental health improvement. These findings suggest that management of preoperative anxiety and depression may improve surgical outcomes in patients undergoing ATFL repair.

## Introduction

Ankle sprains are a common orthopedic injury, accounting for approximately 9–12% of all musculoskeletal injuries (1), with approximately 85% involving the anterior talofibular ligament (ATFL) (2). ATFL injuries frequently result in chronic ankle joint dysfunction, manifesting as persistent pain, mechanical instability, swelling, recurrent sprains, and sensory disturbances (2–4). Surgical intervention is often indicated when the injury is severe or when conservative treatment fails (2, 4–6). Multiple studies have demonstrated that surgical repair of ATFL injuries yields favorable clinical outcomes (3, 4, 7). However, despite these positive surgical results, preexisting mental health conditions may significantly influence postoperative recovery (8–10), suggesting that psychological evaluation may be important in preoperative planning.

Depression and anxiety are prevalent mental health disorders, especially among orthopedic patients, and have consistently been associated with impaired postoperative recovery (9, 11, 12). Furthermore, depressive symptoms are closely associated with delays in postoperative recovery and a decline in quality of life (13). Studies have shown that patients with preoperative anxiety or depressive symptoms tend to exhibit poorer functional outcomes, increased pain, and a higher risk of postoperative complications (14). Harmer et al. reported that anxiety and depressive symptoms are associated with higher rates of infection, revision, and reoperation following total hip or knee arthroplasty (15). Similarly, Nakagawa et al. found that approximately 30% of patients with chronic foot and ankle conditions experience anxiety or depressive symptoms, which are independently associated with more severe pain and a reduced quality of life (16). Moreover, studies have indicated that patients with preoperative psychological symptoms tend to experience poorer postoperative outcomes following ankle surgery (10, 17–19). Wu et al. noted that patients undergoing anterior cruciate ligament (ACL) reconstruction exhibited a higher incidence of postoperative depressive symptoms, which were correlated with suboptimal surgical outcomes (20). Similar findings have been reported among professional athletes with ACL injuries, who are at an increased risk of depression during the recovery period (8). These findings underscore the importance of preoperative mental health screening for patients with patient undergoing orthopedic surgery, to enhance treatment planning and postoperative recovery.

Existing studies primarily focus on comparing the clinical outcomes following ATFL repair and on evaluating the choice of surgical techniques. However, the potential impact of patients’ preoperative mental health status—particularly symptoms of anxiety and depression—on postoperative pain relief, functional recovery, and quality-of-life improvement has not been systematically investigated. Therefore, this study performed a retrospective analysis to investigate the relationship between preoperative anxiety and depressive symptoms and postoperative outcomes, aiming to assess their influence on the clinical efficacy of ATFL repair and to provide evidence for developing individualized treatment strategies. This study hypothesizes that preoperative anxiety and depressive symptoms may significantly influence clinical outcomes after ATFL repair, particularly in terms of pain relief, functional recovery, and improvements in quality of life.

## Patients and methods

### Patients

This study received approval from the Ethics Review Committee of Xi’an Honghui Hospital (No. 2025-KY-115-01). A retrospective analysis was conducted on patients who underwent ATFL reconstruction surgery in the Department of Comprehensive Foot and Ankle Surgery at Xi’an Honghui Hospital between January 2015 and August 2023. All participants provided informed consent, ensuring that the research process adhered to ethical standards.

**Figure 1:**
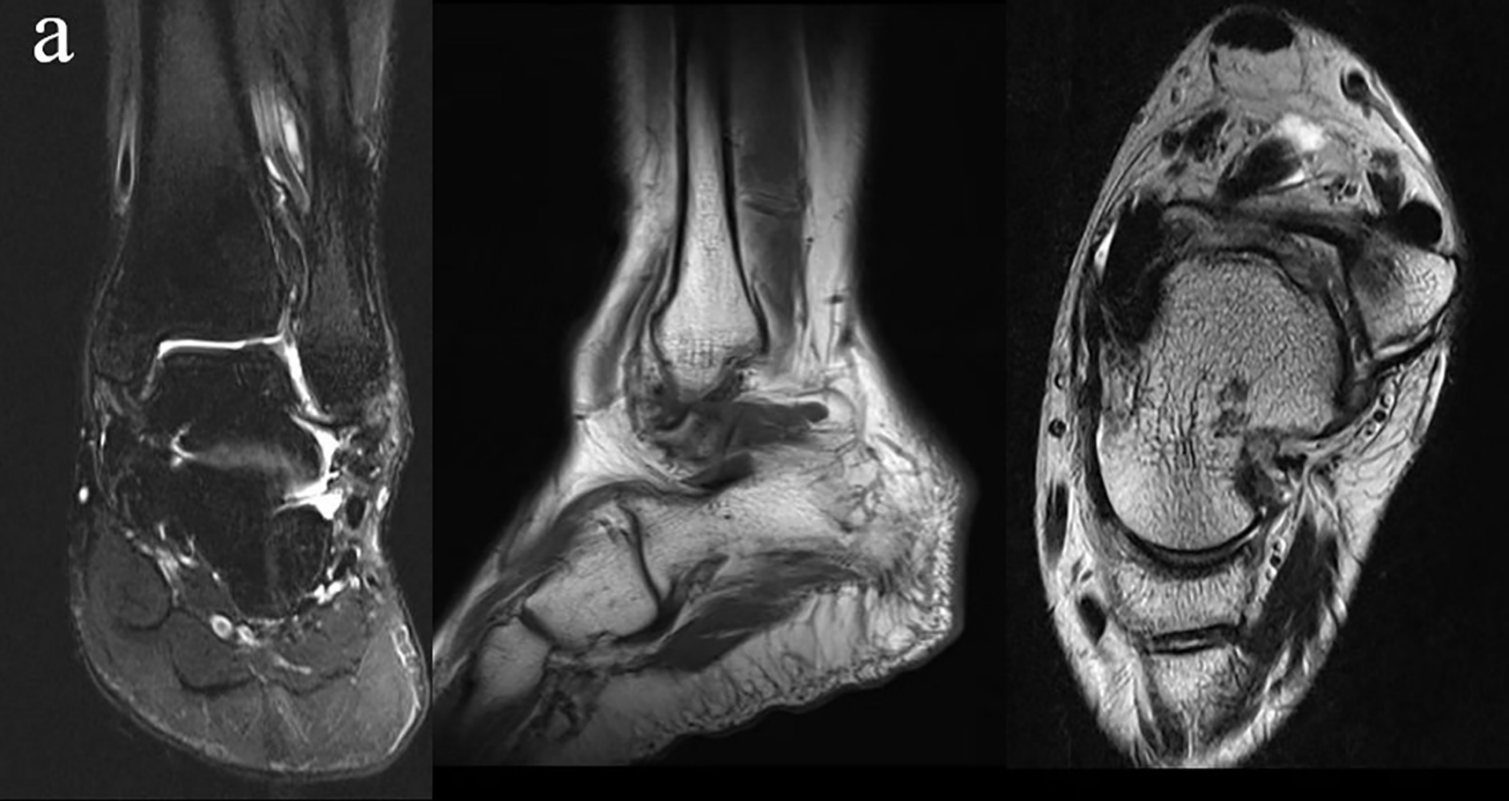
Preoperative MRI reveals an increased signal in the ATFL pathway, which is indicative of an ATFL injury. Additionally, effusion in the ankle joint and swelling of the surrounding soft tissues are observed.

Inclusion criteria: (1) Unilateral ATFL injury (see **Figure 1**); (2) Persistent symptoms after at least 3 months of non-surgical treatment; (3) Age ≥ 18 years; (4) Participants able to independently complete the questionnaire and without severe cognitive impairments. Exclusion criteria: (1) Presence of ankle fractures (confirmed through X-ray or CT imaging); (2) The affected limb has a history of other trauma or surgery; (3) Diagnosis of severe osteoporosis, rheumatic diseases, rheumatoid arthritis, or ankle joint infections; (4) Presence of chronic systemic diseases, including diabetes, hypertension, malignancies, or hepatic/renal dysfunction; (5) The patient has other mental illnesses aside from anxiety/depression.

### Methods

Basic demographic and clinical information—including gender, age, BMI, and disease duration—was collected. Patients were evaluated preoperatively and at the final follow-up using the Hospital Anxiety and Depression Scale (HADS), the American Orthopedic Foot and Ankle Society (AOFAS) Ankle-Hindfoot Score, and the Visual Analog Scale (VAS) for pain. The HADS includes two subscales: Anxiety (HADS-A) and Depression (HADS-D), each comprising seven items rated from 0 to 3, yielding subscale scores ranging from 0 to 21. Scores ≥8 on either subscale indicate the presence of anxiety or depressive symptoms (21). The AOFAS score has a maximum of 100 points and evaluates ankle function through three domains: subjective pain (40 points), objective function (50 points), and alignment (10 points). Higher scores reflect better foot and ankle function (22). The VAS uses a 100 mm horizontal line, where 0 represents “no pain” and 100 represents “worst imaginable pain.” Patients marked their current pain intensity, with higher scores indicating more severe pain (23).

Based on preoperative psychological assessments, patients were divided into two groups. Those with HADS-A and/or HADS-D scores ≥8 were classified into the anxiety/depression group (Group A), while those with scores <8 formed the non-anxiety/depression group (Group B). All ATFL repair procedures were performed by the same team of experienced foot and ankle chief surgeons to ensure consistency in surgical technique. Postoperatively, all patients received standardized rehabilitation protocols to minimize variability in recovery and enable a reliable comparison of outcomes.

### Surgery and rehabilitation

ATFL repair was performed under general anesthesia combined with peripheral nerve block. A longitudinal incision approximately 5 cm in length was made along the anterior border of the distal fibula. The skin, subcutaneous tissue, and deep fascia were incised layer by layer. The fibrous tendon sheath was bluntly dissected to expose the distal fibula. Following incision of the joint capsule, the ATFL was visualized, and synovial hyperplasia and scar tissue were debrided. Intraoperative exploration typically revealed thinning of the ligament substance. A suture anchor loaded with nonabsorbable suture was inserted into the distal fibula, and the ATFL was anatomically repaired using tight suturing. Ankle joint stability was confirmed through intraoperative passive range of motion testing. After adequate irrigation, the wound was closed in layers.

Postoperatively, an ankle brace was applied to maintain the ankle in a neutral dorsiflexion position with 5°–10° of eversion. At 6 weeks after surgery, patients gradually progressed to partial weight-bearing and initiated active dorsiflexion and plantarflexion exercises. Routine follow-up evaluations were conducted at 1, 2, and 3 months postoperatively, and then annually. The recovery of the ligaments and functional progress were monitored through the anterior drawer test, ankle joint range of motion assessment, and ultrasound examination, allowing for the timely detection of potential complications.

### Statistical analysis

All statistical analyses were performed using SPSS software version 25.0 (IBM Corp., Armonk, NY, USA). The Shapiro–Wilk test was used to assess the normality of data distribution for each variable. As the data did not conform to a normal distribution, non-parametric statistical methods were applied. Descriptive statistics are presented as median and interquartile range [M (P25, P75)]. Between-group comparisons were conducted using the Mann–Whitney U test, while gender differences were evaluated using the Chi-square test. Within-group comparisons were assessed using the Wilcoxon signed-rank test. A two-sided P value of < 0.05 was considered statistically significant for all tests.

## Results

### General characteristics of the patients

A total of 91 eligible patients were included in this study, among whom 81 completed follow-up assessments. The median age was 34.00 years (interquartile range [IQR]: 28.00–46.00), median disease duration was 12.00 months (IQR: 5.00–24.00), median BMI was 24.62 kg/m² (IQR: 22.54–25.99), and median follow-up duration was 35.00 months (IQR: 30.00–55.00). Based on preoperative HADS scores, patients were categorized into two groups. Group A (n = 40, 49.38%) included 20 males and 20 females with anxiety/depressive symptoms; Group B (n = 41, 50.62%) included 19 males and 22 females without such symptoms. There were no statistically significant differences in baseline characteristics between the two groups (P > 0.05), as detailed in **Table 1**.

**Table 1:** Comparison of baseline characteristics between Group A and Group B.

Variables	Group A (n=40)		Group B (n=41)		t/z	P
	Male	Female	Male	Female		
Sex	20 (50%)	20 (50%)	19 (46.3%)	22 (53.7%)	0.11	0.74
Age (years)	40 (28.00, 52.00)		34.00 (28.00, 45.00)		-0.55	0.58
Disease duration (months)	15.00 (5.25, 25.50)		10.00 (5.00, 16.50)		-1.38	0.17
BMI (kg/m2)	24.73 (21.50, 25.71)		24.49 (22.54, 26.12)		-0.78	0.43
Follow-up duration (Month)	37.50 (30.25, 48.75)		35.00 (29.00, 70.00)		-0.06	0.96

Group A: Anxiety/Depression group (presence of anxiety/depression symptoms).

Group B: Non-Anxiety/Depression group (absence of anxiety/depression symptoms).

### Effect analysis

A comparison of the VAS, AOFAS, HADS-A, and HADS-D scores before surgery and at the final follow-up for both Group A and Group B demonstrated significant improvements in all indicators at the final follow-up compared to preoperative values (P < 0.001), as shown in **Tables 2** and **3**.

**Table 2:** Comparison of clinical evaluation indicators before surgery and at the final follow-up for patients in Group A.

Variables	Preoperative	Last Follow-up	t	P
VAS	60.00 (60.00, 70.00)	15.00 (5.00, 50.00)	-5.52	<0.001 (0.000)
AOFAS	48.50 (44.25, 56.00)	90.00 (88.00, 97.50)	-5.51	<0.001 (0.000)
HADS-A	8.00 (8.00, 9.00)	2.50 (2.00, 3.75)	-5.53	<0.001 (0.000)
HADS-D	7.00 (6.00, 8.00)	3.00 (2.00, 4.00)	-5.54	<0.001 (0.000)

**Table 3:** Comparison of clinical evaluation indicators before surgery and at the final follow-up for patients in Group B.

Variables	Preoperative	Last Follow-up	t	P
VAS	60.00 (55.00, 65.00)	4.00 (3.00, 4.00)	-5.59	<0.001 (0.000)
AOFAS	53.00 (51.00, 59.00)	100.00 (100.00,100.00)	-5.59	<0.001 (0.000)
HADS-A	4.00 (4.00, 4.50)	1.00 (1.00, 2.00)	-5.66	<0.001 (0.000)
HADS-D	5.00 (4.00, 5.00)	2.00 (2.00, 3.00)	-5.67	<0.001 (0.000)

Furthermore, the comparison of VAS, AOFAS, HADS-A, and HADS-D scores before surgery and at the final follow-up between Group A and Group B revealed that, both preoperatively and at the final follow-up, Group A patients had significantly lower scores compared to Group B patients (P < 0.001), as shown in **Tables 4** and **5**.

**Table 4:** Comparison of clinical evaluation indicators before surgery between patients in Groups A and B.

Variables	Group A (n=40)	Group B (n=41)	t	P
VAS	60.00 (60.00, 70.00)	60.00 (55.00, 65.00)	-3.87	<0.001 (0.000)
AOFAS	48.50 (44.25, 56.00)	53.00 (51.00, 59.00)	-3.68	<0.001 (0.000)
HADS-A	8.00 (8.00, 9.00)	4.00 (4.00, 4.50)	-7.70	<0.001 (0.000)
HADS-D	7.00 (6.00, 8.00)	5.00 (4.00, 5.00)	-6.52	<0.001 (0.000)

Group A: Anxiety/Depression group (presence of anxiety/depression symptoms).

Group B: Non-Anxiety/Depression group (absence of anxiety/depression symptoms).

**Table 5:** Comparison of clinical evaluation indicators at the final follow-up between patients in Groups A and B.

Variables	Group A(n=40)	Group B(n=41)	t	P
VAS	15.00 (5.00, 50.00)	4.00 (3.00, 4.00)	-5.29	<0.001 (0.000)
AOFAS	90.00 (88.00, 97.50)	100.00 (100.00,100.00)	-5.56	<0.001 (0.000)
HADS-A	2.50 (2.00, 3.75)	1.00 (1.00, 2.00)	-5.68	<0.001 (0.000)
HADS-D	3.00 (2.00, 4.00)	2.00 (2.00, 3.00)	-4.32	<0.001 (0.000)

Group A: Anxiety/Depression group (presence of anxiety/depression symptoms).

Group B: Non-Anxiety/Depression group (absence of anxiety/depression symptoms).

A further analysis of the intergroup differences in the improvement of VAS, AOFAS, HADS-A, and HADS-D scores before surgery and at the final follow-up revealed that the improvement in HADS-A and HADS-D scores was significantly greater in Group A than in Group B (P < 0.001). However, no significant differences were found in the improvement of VAS and AOFAS scores between Group A and Group B patients (P > 0.05), as shown in **Table 6**.

**Table 6:** Comparison of improvements in clinical evaluation indicators before surgery and at the final follow-up between patients in Groups A and B.

Variables	Group A(n=40)	Group B(n=41)	t	P
VAS Change	50.00 (40.00, 60.75)	56.00 (47.00, 57.00)	-1.69	0.09
AOFAS Change	42.00 (37.25, 46.75)	42.00 (39.00, 49.00)	-0.87	0.38
HADS-A Change	5.50 (4.00, 7.00)	3.00 (2.00, 3.50)	-6.66	<0.001 (0.000)
HADS-D Change	4.00 (3.00, 5.00)	2.00 (2.00, 3.00)	-4.76	<0.001 (0.000)

Group A: Anxiety/Depression group (presence of anxiety/depression symptoms).

Group B: Non-Anxiety/Depression group (absence of anxiety/depression symptoms).

## Discussion

Clinicians often prioritize techniques for repairing ATFL injuries and selecting appropriate grafts (4, 24, 25), while overlooking the mental health aspects of patients. Nakagawa et al. reported that approximately 30% of patients with chronic foot and ankle disorders experience anxiety or depression (16). Several studies have demonstrated that patients exhibiting preoperative anxiety or depression symptoms tend to have poorer clinical outcomes following ankle surgery compared to those without such symptoms (19, 26, 27). However, research on the mental health status of patients with ATFL injuries remains scarce, particularly regarding the influence of preoperative anxiety or depression on surgical outcomes. Thus, this study conducted preoperative mental health screenings for patients with ATFL injuries and evaluated the effect of preoperative anxiety and depression symptoms on surgical outcomes.

In this study, following ATFL injury reconstruction surgery, the VAS, AOFAS, HADS-A, and HADS-D scores at the final follow-up were significantly improved compared to preoperative scores, regardless of whether the patient had preoperative anxiety/depression symptoms, indicating the substantial efficacy of the surgery. ATFL injuries can lead to chronic ankle joint disability, including persistent pain, functional instability, swelling, recurrent sprains, and sensory impairments, among other symptoms (2–4). Numerous studies on ATFL injury reconstruction surgery have demonstrated significant improvements in VAS and AOFAS scores at the final follow-up compared to preoperative levels (2, 3, 5). Additionally, relevant research indicates that, regardless of preoperative mental health status, patients undergoing total ankle arthroplasty or ankle arthrodesis experienced significant improvements in mental health, foot function, and pain at the final follow-up (19, 27), which aligns with the findings of this study.

However, it is noteworthy that this study also found that patients with preoperative anxiety/depression symptoms had significantly lower VAS, AOFAS, HADS-A, and HADS-D scores at both the preoperative and final follow-up compared to those without preoperative anxiety/depression symptoms. Multiple studies have demonstrated an interaction between anxiety/depression and pain perception, as well as functional limitations (16, 28, 29). Previous studies have confirmed that patients with anxiety/depressive symptoms are more likely to exhibit higher pain sensitivity (30, 31), and the increase in pain perception may further exacerbate the limitations in functional activities. Conversely, pain perception and activity limitations further affect the patients’ mental health. Thus, preoperative anxiety/depression symptoms lead to poorer preoperative pain and functional scores, while pain and functional outcomes at the final follow-up are negatively influenced by the preoperative mental state, resulting in poorer clinical outcomes and ultimately affecting patients’ mental health.

This phenomenon may be related to the effects of anxiety and depression on physiological factors, such as immune function and hormone levels, in patients. Previous studies have shown that anxiety and depressive symptoms may alter the endocrine system’s response, including increased cortisol levels, thereby affecting patients’ pain perception and recovery of physical function (32, 33). Cortisol, as a stress hormone, plays a key role in responding to physical stress and inflammatory reactions. Research has found that prolonged elevated cortisol levels may lead to an enhanced inflammatory response, thereby exacerbating pain and functional impairments (34). Moreover, anxiety and depressive symptoms may also affect the immune system, resulting in abnormal immune responses and further delaying postoperative recovery (35). Therefore, mental health issues may directly affect patients’ pain perception and functional recovery via physiological pathways.

Additionally, this study conducted a comparative analysis of the changes in VAS, AOFAS, HADS-A, and HADS-D scores between the two patient groups at the preoperative and final follow-up stages. The results revealed that patients with preoperative anxiety/depression symptoms exhibited significantly greater improvements in HADS-A and HADS-D scores compared to those without anxiety/depression symptoms. However, no significant differences were observed between the groups in terms of VAS and AOFAS score improvements. We propose that surgical intervention not only alleviated pain and enhanced foot function but also, to some extent, promoted emotional recovery in patients with preoperative psychological distress. Due to the higher baseline psychological scores in the anxiety/depression group, their postoperative improvement was more pronounced, but this did not affect their final functional recovery. In other words, while patients in the preoperative anxiety/depression group showed more significant mental health improvements, both groups experienced similar pain relief and functional recovery outcomes, suggesting that surgery offers consistent functional benefits regardless of mental health status. Similar findings have been reported in patients undergoing hallux valgus surgery, where two years after surgery, patients with psychological distress demonstrated significantly greater mental health improvement, but the changes in VAS and AOFAS scores were comparable to those without psychological distress (36). Additionally, studies on foot and ankle surgery indicated that patients with higher preoperative anxiety scores experienced greater improvements in anxiety scores, but there were no significant differences in pain and functional recovery compared to patients with lower preoperative anxiety scores (37). These results further validate our conclusion.

However, the study has several limitations that should be addressed in future research. First, the sample size of this study is relatively small, which may affect the generalizability of some of the results. We recommend that future studies use larger sample sizes to ensure that the research results have stronger external validity and generalizability. Second, this is a single-center, retrospective study with all patients drawn from the same tertiary, specialized orthopedic hospital, which may limit the applicability of the results to other regions or medical institutions. Third, the study primarily focused on anxiety and depression as mental health indicators, without considering other psychological factors that could influence surgical outcomes, such as emotional regulation and stress levels. Future studies could incorporate additional dimensions of mental health into the assessment to thoroughly analyze the impact of different psychological factors on surgical outcomes. Finally, although this study analyzed changes in clinical outcomes, it did not fully account for potential confounding factors, such as individual patient differences, cultural background, and socioeconomic status, which could significantly influence postoperative recovery.

## Conclusion

ATFL reconstruction surgery significantly enhances patients’ pain relief, functional recovery, and overall satisfaction. Patients with preoperative anxiety/depression symptoms demonstrated lower clinical evaluation scores at both the preoperative and final follow-up stages compared to those without such symptoms. Despite worse postoperative outcomes in patients with preoperative anxiety/depression symptoms, both groups exhibited similar improvements in pain relief and functional recovery. Furthermore, patients with preoperative anxiety/depression symptoms showed more substantial improvements in anxiety and depression levels at the final follow-up. These findings highlight the importance of preoperative mental health assessment and timely psychological interventions to optimize surgical outcomes.

## Data Availability

The raw data supporting the conclusions of this article will be made available by the authors, without undue reservation.
